# Comparative Analysis of Rhizosphere Microbiomes of Southern Highbush Blueberry (*Vaccinium corymbosum* L.), Darrow’s Blueberry (*V. darrowii* Camp), and Rabbiteye Blueberry (*V. virgatum* Aiton)

**DOI:** 10.3389/fmicb.2020.00370

**Published:** 2020-03-12

**Authors:** Jiangang Li, Olga V. Mavrodi, Jinfeng Hou, Chazden Blackmon, Ebrahiem M. Babiker, Dmitri V. Mavrodi

**Affiliations:** ^1^Key Laboratory of Soil Environment and Pollution Remediation, Institute of Soil Science, Chinese Academy of Sciences, Nanjing, China; ^2^Department of Cell and Molecular Biology, School of Biological, Environmental, and Earth Sciences, The University of Southern Mississippi, Hattiesburg, MS, United States; ^3^South Mississippi Branch Experiment Station, Mississippi State University, Poplarville, MS, United States; ^4^USDA-ARS Southern Horticultural Research Laboratory, Poplarville, MS, United States

**Keywords:** *Vaccinium*, blueberry, rhizosphere, microbiome, 16S, ITS, 18S

## Abstract

Plants are inhabited by millions of parasitic, commensal, and mutualistic microorganisms that coexist in complex ecological communities, and profoundly affect the plant’s productivity, health, and capacity to cope with environmental stress. Therefore, a better understanding of the rhizosphere microbiome may open a yet untapped avenue for the rational exploitation of beneficial plant–microbe interactions in modern agriculture. Blueberries encompass several wild and cultivated species of shrubs of the genus *Vaccinium* that are native to North America. They are grown commercially for the production of fruits, which are considered a health food due to the rich content of minerals, trace elements, and phenolic compounds with antioxidant, antitumor, and anti-inflammatory properties. Despite a long history of breeding and extensive commercial use, remarkably little is known about the composition and function of the blueberry root microbiome. To address this gap, we employed molecular approaches to characterize and compare microbial communities inhabiting the roots of rabbiteye blueberry (*Vaccinium virgatum*), Darrow’s blueberry (*Vaccinium darrowii*), and southern highbush blueberry (SHB; an interspecific hybrid of *Vaccinium corymbosum* and *V. darrowii*). Our results revealed that these plant species share a common core rhizobiome, but at the same time differ significantly in the diversity, relative abundance, richness, and evenness of multiple groups of prokaryotic and eukaryotic microorganisms. Although the host signature effects were especially pronounced at the plant species level, we also observed genotype-level variations in the distribution of specific microbial taxa, which suggests that the assembly of the blueberry microbiome is shaped by the plant genotype and modifications associated with the domestication and breeding of members of the *Vaccinium* genus. We also demonstrated that the studied *Vaccinium* species differ in the abundance of beneficial rhizobacteria and ericoid mycorrhizal fungi, which play a vital role in their adaptation to soils with low pH and slow turnover of organic matter.

## Introduction

Plants are meta-organisms that are inhabited by millions of parasitic, commensal, and mutualistic microorganisms that coexist in complex ecological communities (Vandenkoornhuyse et al., 2015). The plant microbiome profoundly affects the development, health, productivity, and capacity of plants to cope with abiotic and biotic stresses and represents an area of active ongoing research. Most plant-associated microbes are found in contact with, or in immediate proximity to plant roots, where they form part of an extended food web driven by the release of plant rhizodeposits, or exudates (Sørensen and Sessitsch, 2006). Plants and their root-associated microbiome (rhizobiome) have both evolved to use their close association for the mutual benefit. The host plant provides rhizosphere microorganisms with a carbon-rich niche formed by the secretion of exometabolites into the soil that directly surrounds roots (Bulgarelli et al., 2012, 2015; Badri et al., 2013). In return, members of the rhizosphere and root microbiota supply plants with macro- and micronutrients, stimulate organ development, suppress pathogens, and modulate levels of stress phytohormones (Bulgarelli et al., 2015).

The makeup and function of the rhizobiome are strongly influenced by soil properties and by the presence and composition of rhizodeposits (Peiffer et al., 2013, Canarini et al., 2019). Root exudates contain a complex mixture of high- (lysates, mucilages, proteins), and low-molecular-weight (carbohydrates, amino acids, organic and fatty acids, phenolics, sterols) metabolites that supply rhizobacteria with carbon, nitrogen, and energy for growth. The amounts and patterns of rhizodeposition change depending on the plant growth stage, environmental factors, and abiotic stressors (Sasse et al., 2018). The profiles of exudation also vary substantially within and between plant species and it is thought that these differences drive the recruitment of specific taxa from the microbial seed bank of the soil (Lareen et al., 2016; Beattie, 2018). Consistent with the idea that plants actively select and shape their root microbiota, comparative analysis revealed significant variations in the composition of root-associated microbial communities across 30 species of angiosperm plants (Fitzpatrick et al., 2018). Interestingly, the structure and function of the rhizobiome are also significantly affected by the process of domestication, and commercial varieties of barley, maize, lettuce, beet, and agave harbor rhizosphere communities that differ substantially from their counterparts in the closely related species of wild plant (Zachow et al., 2014; Bulgarelli et al., 2015; Cardinale et al., 2015; Szoboszlay et al., 2015; Coleman-Derr et al., 2016). Although the available information is limited, several studies reported that domestication changes the rhizomicrobial diversity and affects the association with beneficial mycorrhizal fungi and nitrogen-fixing rhizobia (Perez-Jaramillo et al., 2016, 2018). Therefore, a better understanding of the impact of plant breeding on the composition and function of the rhizosphere microbiome may open a yet untapped avenue for the rational exploitation of beneficial plant–microbe interactions in modern agriculture.

Blueberries encompass several wild and cultivated species of shrubs of the genus *Vaccinium* L. that are native to eastern North America (Camp, 1945). They are grown commercially for the production of fruits, which are considered a health food due to the rich content of minerals, trace elements, and phenolic compounds with antioxidant, antitumor, and anti-inflammatory properties (Wang et al., 1997; Lobos and Hancock, 2015; Massarotto et al., 2016). The breeding and selection of blueberries began in the early 1900s (Coville, 1937), and three species, Tetraploid lowbush *Vaccinium angustifolium* Aiton (2*n* = 4× = 24), tetraploid highbush *Vaccinium corymbosum* L. (2*n* = 4× = 48), and hexaploid rabbiteye *Vaccinium virgatum* Aiton (2*n* = 6× = 72), constitute the backbone of the current commercial cultivars (Chavez and Lyrene, 2009). Over the past 70 years, the acreage of the highbush varieties has expanded dramatically due to the introduction of the southern highbush blueberry (SHB) with lower chilling requirements (Retamales and Hancock, 2018). The development of SHB cultivars was initiated by crosses between the tetraploid NHB *V. corymbosum* L. to Florida’s native diploid blueberry species *Vaccinium darrowii* Camp, but later, native *V. angustifolium* and *V. virgatum* were introduced into breeding programs (Sharpe and Darrow, 1959). These efforts helped to introduce novel adaptation genes and led to the release of several commercial cultivars with improved tolerance to higher soil pH and drought (Finn et al., 1993; Nunez et al., 2015). However, despite significant progress, the expansion of SHB in the Gulf Coast region of the United States is still challenged by fluctuations in temperature, rainfall patterns, UV levels, elevated soil pH, and drought (Lobos and Hancock, 2015).

The ability of the rhizosphere microorganisms to influence plant susceptibility to diseases and fitness in response to water stress, salinization, and soil pollution prompted detailed microbiome studies in numerous crop species (Raaijmakers et al., 2009; Edwards et al., 2015; Mahoney et al., 2017; Pfeiffer et al., 2017). In contrast, most relevant studies in blueberries employed traditional culture-based approaches and focused primarily on the association of *Vaccinium* spp. with ericoid mycorrhizae. The only comprehensive microbiome study focused on the effect of cultural practices on the structure of rhizosphere microbial communities of wild blueberry *V. angustifolium* grown in managed and forest sites in Nova Scotia, Canada (Yurgel et al., 2017, 2018). Hence, despite a long history of breeding and extensive commercial use, remarkably little is known about the composition and function of blueberry rhizobiome. We hypothesized that the domestication and long history of breeding introduced changes in the structure of blueberry rhizobiome. We tested this hypothesis by comparing the diversity and abundance of bacteria, fungi, and eukaryotic organisms in microbiomes associated with roots of two genotypes each of the SHB (*V. corymbosum* Camp), Darrow’s blueberry (*V. darrowii* Camp), and rabbiteye blueberry (*V. virgatum* Aiton).

## Materials and Methods

### Plant Growth Conditions

The study employed three different *Vaccinium* species, including *V. virgatum* Aiton (Vg) (2*n* = 6× = 72), *V. corymbosum* L. (SHB) (2*n* = 4× = 48), and *V. darrowii* Camp (Vd) (2*n* = 2× = 24) (Table 1). The *V. virgatum* species was represented by breeding selections MS 1089 and MS 1408, while *V. corymbosum* was represented by MS 2337 and MS 2276. *V. darrowii* was represented by the wild-type clone B0008 and breeding selection MS 2230. Plants were grown in a greenhouse in a mixture of pine bark mulch and sand (1:1, v/v) with pH 5.2. Briefly, rooted cuttings of all genotypes were transplanted into 1-gallon pots filled with the potting mix and maintained in a greenhouse under 16 h photoperiod at 22/18°C day/night temperature and drip-irrigated daily with 300 mL of water per pot. Every 15 weeks the pots were fertilized with 1 g of the Osmocote^®^ 14-14-14 slow-release fertilizer (ICL Specialty Fertilizers—North America, Dublin, OH, United States). After about 48 weeks of growth, the plants were transferred in a laboratory, carefully uprooted, and processed for the isolation of rhizosphere soil DNA. The pH of potting mix was monitored with a B-213 Twin pH meter (Horiba Instruments, Irvine, CA, United States).

**TABLE 1:** Description of species and cultivars of *Vaccinium* used in the study.

Treatment	*Vaccinium* species	Sub-treatment	*Vaccinium* genotypes (breeding selections)	Pedigree	Blueberry type
Vg	*V. virgatum* (2*n* = 6× = 72)	Vg1	MS 1089	Baldwin/96-6	Rabbiteye
		Vg2	MS 1408	T366/MS 635	
SHB	*V. corymbosum* (interspecific hybrid, 2*n* = 4× = 48)	SHB1	MS 2337	Pearl/MS 1387	Southern highbush
		SHB2	MS 2276	MS 771/Abundance	
Vd	*V. darrowii* (2*n* = 2× = 24)	Vd1	Clone B0008	Selection from the wild in Florida	Darrow’s
		Vd2	MS 2230	Aromi Sunshine/NJ8810-13	

### Extraction of Rhizosphere Soil DNA and Sequencing of 16S rRNA, ITS, and 18S rRNA Amplicons

Partial root systems were cut from blueberry plants (*n* = 6 per *Vaccinium* genotype), excess soil was removed, and 1 g of excised roots was placed into 50 mL Falcon tubes. Each tube was filled with 20 mL of sterile water and rhizosphere soil was dislodged by a combination of vortexing and treatment in an ultrasonic bath. The resultant soil suspensions were used for the extraction of rhizosphere soil DNA with a DNeasy PowerSoil kit (Qiagen, Germantown, MD, United States). The concentration of the purified DNA was measured using a DNA Quantification kit (Bio-Rad, Hercules, CA, United States) by measuring fluorescence at 460 nm with a Synergy 2 microplate reader (BioTek Instruments, Winooski, VT, United States). The absence of PCR inhibitors in the extracted DNA was verified by PCR with the DreamTaq DNA polymerase (Thermo Fisher Scientific, Waltham, MA, United States) and 16S rRNA-specific universal eubacterial primers 8F and 1492R (Weisburg et al., 1991). The amplification was performed as described by Mavrodi et al. (2018). The samples of purified rhizosphere DNA were shipped for analysis to the Center for Comparative Genomics and Evolutionary Bioinformatics at Dalhousie University^1^. Barcoded amplicons were generated by PCR with the high-fidelity Phusion DNA polymerase (Thermo Fisher Scientific) and primers targeting the V6–V8 region of bacterial 16S rRNA (forward primer B969F: ACGCGHNRAACCTTACC; reverse primer BA1406R: ACGGGCRGTGWGTRCAA) (Comeau et al., 2011), the internal transcribed spacer (ITS) 2 region of fungi [forward primer ITS86(F): GTGAATCATCGAATCTTTGAA; reverse primer ITS4(R): TCCTCCGCTTATTGATATGC] (Op De Beeck et al., 2014), and the V4 region of eukaryotic 18S rRNA (forward primer: CYGCGGTAATTCCAGCTC; reverse primer: AYGGTATCTRATCRTCTTYG). The purification of amplicons, library preparation, and its sequencing on a MiSeq instrument (Illumina, San Diego, CA, United States) using the MiSeq v3 chemistry (2 × 300 bp) were performed as described by Comeau et al. (2017).

### Bioinformatics Analyses

The Illumina sequence data were processed using the Microbiome Helper pipeline (Comeau et al., 2017). The sequence quality was confirmed with the FastQC toolkit^2^, after which the forward and reverse reads were merged with PEAR v 0.9.10 (Zhang et al., 2014) followed by filtered out the low-quality and chimeric reads with FASTX-Toolkit v 0.0.14^3^ and USEARCH v 6.1 (Edgar et al., 2011), respectively. Operational taxonomic units (OTUs) were characterized by matching to the RDP 16S rRNA database (11 release) (Cole et al., 2014) and the UNITE database (12_11 release) of the fungal ITS sequences (Abarenkov et al., 2010), and SILVA dataset of 18S rRNA sequences (Yilmaz et al., 2014). Reads were subsequently mapped back to OTUs to determine the OTU abundance for each sample, and differences in the community composition/structure in each sample were analyzed with the Quantitative Insights Into Microbial Ecology (QIIME) package (Caporaso et al., 2010). Any OTUs that constituted less than 0.1% of the total OTUs were removed, and the data were normalized by the total sum normalization (TSS) with square root transformation. Differences in alpha diversity between the species of *Vaccinium* were determined by Duncan’s new multiple range test (*P* < 0.05). Differences in the diversity and OTU abundance between genotypes of the same *Vaccinium* species were determined by the two-sample *t*-test (*P* < 0.05) or by the Wilcoxon rank-sum test (*P* < 0.05) and visualized in the heat tree format using Metacoder (Foster et al., 2017). The R package Phyloseq (McMurdie and Holmes, 2013) was used to generate non-metric multidimensional scaling (NMDS) ordination (*K* = 2) based on Bray–Curtis dissimilarity matrices to visualize the most abundant phyla from different *Vaccinium* species and genotypes. The amount of variation in the composition of bacterial, fungal, and eukaryotic communities that could be explained by *Vaccinium* species and genotypes was estimated by the permutational multivariate analysis of variance (PERMANOVA) following the calculation of a Bray–Curtis dissimilarity matrix with 999 permutations. Network analyses and identification of differentially enriched microbial taxa using the linear discriminant analysis effect size (LEfSe) method were carried out in Calypso (Zakrzewski et al., 2017).

### Community Level Physiological Profiling (CLPP)

In order to perform the CLPP analysis, 1–3 g of plant roots were gently shaken to remove excess soil and placed into 50 mL conical centrifuge tubes. Nine parts of phosphate-buffered saline (PBS) were added to each tube, and root-associated bacteria were dislodged by vortexing and treatment in a sonicating bath (1 min each). All root washes were further diluted 10-fold with PBS and inoculated into EcoPlates (100 μL per well) containing 31 different carbon sources (Biolog, Hayward, CA, United States). The inoculated EcoPlates were incubated for a week at 23°C in the dark, and patterns of the C source utilization were scored daily by measuring absorbance at 590 nm with a Synergy 2 microplate reader (BioTek Instruments). Functional diversity and CLPP similarity indices were calculated based on readings obtained on the sixth day of incubation. The entire experiment was repeated twice.

### Data Availability

Sequences generated in this project were deposited in the NCBI sequence read archive under accession numbers PRJNA577971 and PRJNA578171.

## Results

### Composition of Rhizobiomes Associated With *V. virgatum*, *V. corymbosum*, and *V. darrowii*

**FIGURE 1:**
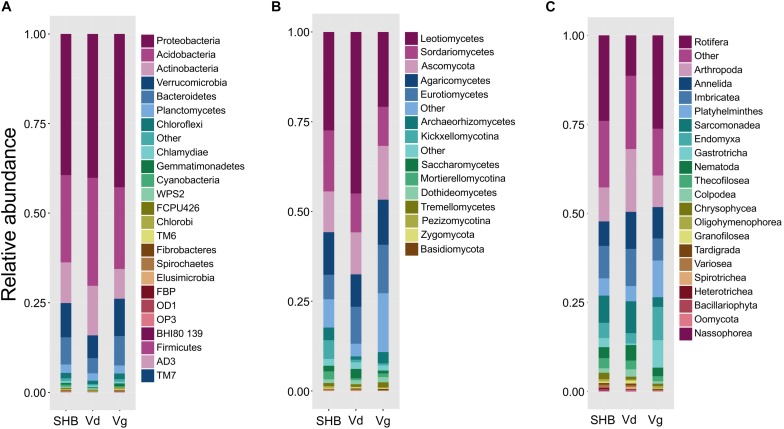
The relative abundance of major bacterial (phylum level) **(A)**, fungal (class level) **(B)**, and non-fungal eukaryotic (class level) **(C)** taxa present in the rhizosphere of different blueberry species.

The rhizosphere communities were characterized via high-throughput sequencing of 16S, ITS2, and 18S amplicons generated using DNA extracted from the rhizosphere of two genotypes each of *V. virgatum* (Vg), *V. corymbosum* (SHB), and *V. darrowii* (Vd) species of blueberry (Table 1). In order to profile the bacterial part of the rhizobiome, pools of 16S amplicons were processed to remove low-quality reads, chimeras, and samples with a low depth of coverage. The resultant dataset of 2,096,766 high-quality reads (median reads per sample = 60,990) was rarified to an even depth of 21,000 reads and binned into OTUs at 97% sequence identity. We observed OTUs from 15 bacterial phyla with an average relative abundance of above 0.1%, and seven of these phyla (*Proteobacteria*, *Acidobacteria*, *Actinobacteria*, *Verrucomicrobia*, *Bacteroidetes*, *Planctomycetes*, and *Chloroflexi*) collectively accounted for 96.7% of all sequencing reads (Figure 1A). Despite the fact that *Vaccinium* species harbored overall similar rhizobacterial communities, we observed several differences in the abundance of individual phyla. For example, samples from SHB were characterized by higher levels of *Cyanobacteria*, whereas Vd had a higher abundance of *Actinobacteria*, *Acidobacteria*, and *Gammaproteobacteria* (Kruskal–Wallis, *P*_FDR_ < 0.05). In contrast, Vg had a higher abundance of *Beta-* and *Deltaproteobacteria, Gemmatimonadetes, Chlorobi, Bacteroidetes, Verrucomicrobia, Fibrobacteres*, and *Spirochaetes* (Kruskal–Wallis, *P*_FDR_ < 0.05). Genotypes of the same *Vaccinium* species were not significantly different (*P*_FDR_ > 0.05) in the relative abundance of prevalent bacterial phyla (data not shown). Core microbiome analysis with the detection threshold of 70% sample prevalence and minimum relative abundance of 0.01% identified several bacterial families, including *Hyphomicrobiaceae, Acetobacteriaceae, Rhodospirillaceae, Koribacteriaceae, Sinobacteriaceae, Acidobacteriaceae, Chitinophagaceae, Solibacteriaceae*, and *Opitutaceae* (Supplementary Figure S1A). Many of these dominant core microbiome families belonged to *Alphaproteobacteria* (especially *Rhizobiales*), as well as to *Acidobacteria* and *Verrucomicrobia.*

The analysis of the fungal communities was performed using a dataset of 1,770,655 high-quality ITS reads (median reads per sample = 50,066), which was obtained after the removal of low-quality and chimeric sequences, as well as samples with the low depth of coverage. The ITS dataset was further rarified to an even depth of 8,000 reads and binned into OTUs at 97% sequence identity. At the phylum level, most of the OTUs were classified as *Ascomycota* (72.4%) and *Basidiomycota* (12.4%), whereas at the class level the communities were dominated by *Leotiomycetes* (30.6%), *Sordariomycetes* (12.9%), *Agaricomycetes* (11.2%), *Eurotiomycetes* (10.4%), *Archaeorhizomycetes* (2.5%), *Saccharomycetes* (1.6%), *Dothideomycetes* (1.2%), and *Tremellomycetes* (1.0%) (Figure 1B). Several groups of these fungi varied significantly between the studied blueberry species, including *Zygomycota, Rozellomycota*, *Kickxellomycotina*, and *Archaeorhizomycetes*, which were more abundant in the rhizosphere of *V. corymbosum* (SHB) (Kruskal–Wallis, *P*_FDR_ < 0.05). In contrast, *V. darrowii* (Vd) had higher abundance of *Ascomycota*, *Leotiomycetes*, and *Saccharomycetes*, whereas samples from *V. virgatum* (Vg) had higher abundance of *Lecanoromycetes* (Kruskal–Wallis, *P*_FDR_ < 0.05). The core microbiome analysis identified *Leotiaceae*, *Trichocomaceae*, *Hypocreaceae*, *Hyaloscyphaceae*, *Herpotrichiellaceae*, *Archaeorhizomycetaceae*, *Saccharomycetaceae*, *Mortierellaceae*, *Cryptococcaceae*, *Cordycipitaceae*, and *Amphisphaeriaceae* as the dominant fungal families of the *Vaccinium* rhizobiome (Supplementary Figure S1B). Interestingly, the first and third most abundant fungal genera of the core microbiome were *Pezoloma* and *Hyaloscypha*, which are prominent ericoid mycorrhizal symbionts of the *Ericaceae* plants, including species of the genus *Vaccinium*.

As with the 16S and ITS amplicons, the 18S-based analysis of eukaryotic communities was initiated by the removal of low-quality sequences, chimeras, and low-coverage samples followed by binning into OTUs at 97% identity. The resultant dataset of 443,016 high-quality reads (median reads per sample = 13,262) was processed further to filter out the unassigned OTUs and plant-derived *Archaeplastida* sequences and then normalized to a depth of 1,459 reads. A largest proportion of 18S OTUs belonged to fungi (54.9%), *Metazoa* (26.1%), *Cercozoa* (11%), and *Alveolata* (1.0%) (data not shown). In agreement with results of the ITS profiling, most fungal reads were belonged to *Ascomycota*, followed distantly by *Basidiomycota*, *Kickxellomycotina*, and *Chytridiomycota*. The non-fungal OTUs collectively accounted for 42.8% of the 18S reads and were dominated by *Rotifera* (9.4%), *Arthropoda* (6.6%), *Cryptomycota* (5.3%), *Platyhelminthes* (3.5%), *Imbricatea* (3.3%), *Annelida* (3.2%), *Endomyxa* (3.0%), *Sarcomonadea* (2.5%), *Gastrotricha* (2.0%), and *Nematoda* (1.3%) (Figure 1C). The core part of the eukaryotic rhizobiome was dominated by several families of *Cercozoa*, including *Trinematidae*, *Euglyphidae*, *Leptophryidae*, *Vampyrellidae*, *Sandonidae*, *Allapsidae*, and *Cercomonadidae* (Supplementary Figure S1C). Other dominant core microbiome groups included arthropods (*Arachnida* and *Maxillopoda*), chrysophytes, and catenulid flatworms. Although the studied *Vaccinium* species harbored similar eukaryotic rhizosphere communities, we observed significantly higher levels of *Sarcomonadea* in *V. darrowii* (Vd), while *Rotifera*, *Endomyxa*, and *Gastrotricha* were more abundant in Vg samples (Kruskal–Wallis, *P*_FDR_ < 0.05).

### Diversity of Rhizosphere Microbial Communities

**FIGURE 2:**
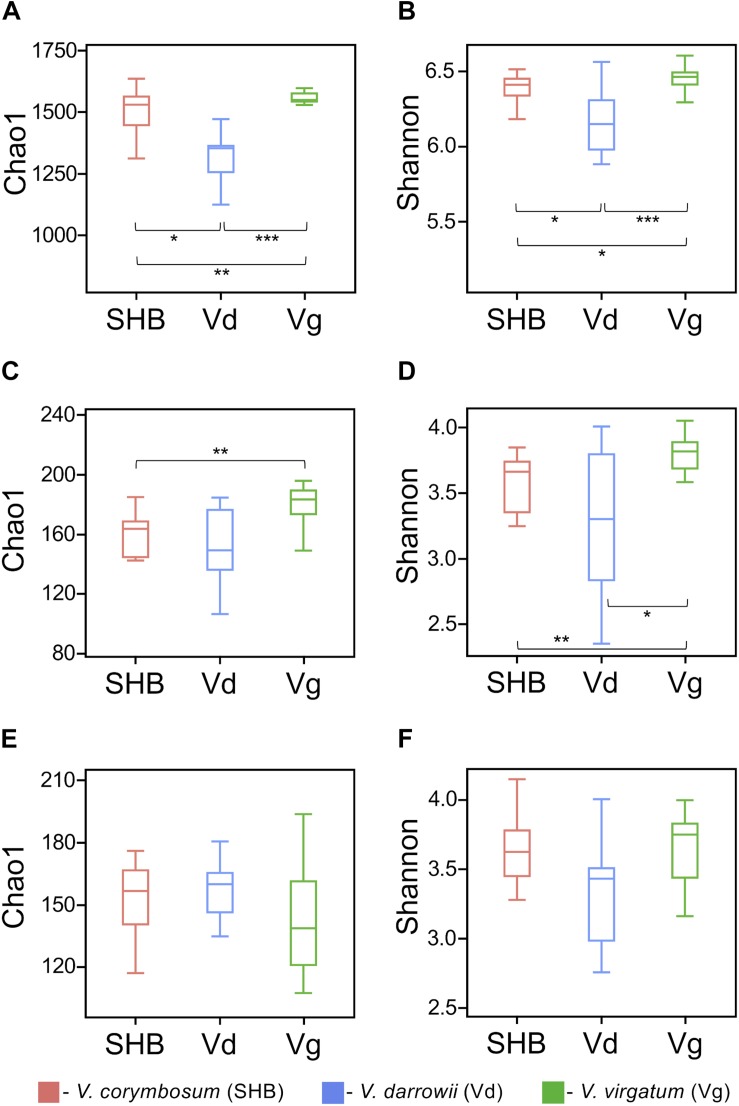
Alpha diversity (Chao1 and Shannon indices) of bacteria **(A,B)**, fungi **(C,D)**, and non-fugal eukaryotes **(E,F)** in the rhizosphere of studied blueberry species. Significant differences between *V. corymbosum*, *V. darrowii*, and *V. virgatum* are indicated in each figure panel (*P < 0.05, **P < 0.01, ***P < 0.001).

The alpha diversity of rhizosphere microbial communities was assessed by calculating Chao1 and Shannon indices. The Chao1 index is as an abundance-based coverage estimator of richness, whereas the Shannon index takes into account both richness and evenness. For rhizobacteria, both metrics indicated significant differences between the studied *Vaccinium* species, with SHB and Vg harboring communities of significantly higher diversity compared to Vd (Kruskal–Wallis; *P* < 0.05) (Figures 2A,B). The comparison of Chao1 and Shannon indices also revealed significant variation in the richness and evenness of fungal communities, which were higher in *V. virgatum* (Vg) compared to *V. corymbosum* (SHB) and *V. darrowii* (Vd) (Figures 2C,D). In contrast, the eukaryotic alpha diversity estimated using Chao1 and Shannon metrics showed that differences between the *Vaccinium* species were not significant (Kruskal–Wallis; *P* < 0.05) (Figures 2E,F). Similarly, no significant differences in the alpha diversity of rhizosphere-dwelling bacteria, fungi, and eukaryotes were detected between different genotypes of the same *Vaccinium* species (Wilcoxon rank-sum test, *P* > 0.05).

**FIGURE 3:**
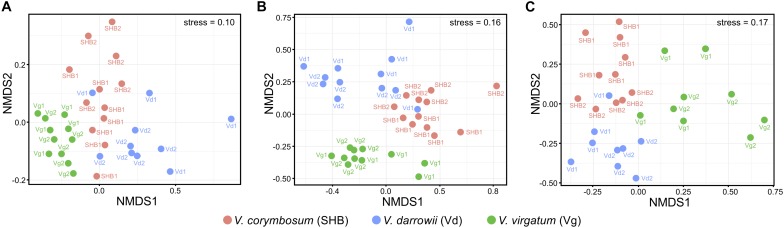
Ordination of bacterial **(A)**, fungal **(B)**, and eukaryotic **(C)** components of the blueberry rhizobiome by the non-metric multidimensional scaling (NMDS) analysis based on Bray–Curtis dissimilarity matrices. Samples from different *Vaccinium* genotypes are color-coded and labeled as follows: Vg1, *V. virgatum* MS 1089; Vg2, *V. virgatum* MS 1408; SHB1, *V. corymbosum* MS 2337; SHB1, *V. corymbosum* MS 2276; Vd1, *V. darrowii* B0008; Vd2, *V. darrowii* MS2230.

The NMDS analysis based on Bray–Curtis dissimilarities revealed that bacterial and fungal communities of the studied *Vaccinium* species clustered distinctively in the ordination space (Figure 3). These results were confirmed by the analysis of similarity (ANOSIM) test, which showed significant differences in the composition of bacterial (*R* = 0.605, *P* = 0.001), fungal (*R* = 0.613, *P* = 0.001), as well as eukaryotic (*R* = 0.548, *P* = 0.001) communities across the three *Vaccinium* species (Supplementary Table S1). Finally, the permutational multivariate ANOVA (Adonis) also demonstrated that bacterial (*R*^2^ = 0.33, *P* < 0.001), fungal (*R*^2^ = 0.28, *P* < 0.001), and eukaryotic (*R*^2^ = 0.22, *P* < 0.001) components of the rhizobiome were significantly differentiated by *Vaccinium* species. Interestingly, some variation in the composition of rhizobacterial and fungal communities was also significantly associated with genotypes of *Vaccinium* species (Adonis test: bacteria *R*^2^ = 0.16, *P* < 0.001; fungi *R*^2^ = 0.26, *P* < 0.001; eukaryotes *R*^2^ = 0.42, *P* < 0.001). A particularly pronounced genotype effect was observed in *V. darrowii*, where the Vd1 and Vd2 bacterial and fungal communities were clearly separated by results of the ANOSIM test (bacteria *R* = 0.70, *P* = 0.008; fungi *R* = 0.96, *P* = 0.004; eukaryotes *R* = 0.69, *P* = 0.014) (Supplementary Table S1).

Finally, we compared the catabolic potential of rhizosphere microbial communities based on the utilization of 31 different carbon substrates present in Biolog EcoPlates. Results of the principal component analysis (PCA) readily distinguished catabolic profiles of the three *Vaccinium* rhizobiomes, with the first two principal components (PC1 and PC2) accounting for 45 and 19% of the total variance (Supplementary Figure S2A). The hierarchical clustering analysis of absorbance values suggested a broader range of metabolic activity in Vg and SHB samples, which resulted in the utilization (OD_590_ >0.1) of 27 different carbon sources (Supplementary Figure S2B). In contrast, only 21 substrates were metabolized in EcoPlates inoculated with the Vd root washes. Rhizosphere microorganisms from the three species of *Vaccinium* actively consumed several carbohydrates (D-galacturonic acid, *N*-acetyl-D-glucosamine, D-mannitol, D-cellobiose, D-glucosaminic acid), L-asparagine, L-phenylalanine, pyruvic acid methyl ester, γ-hydroxybutyric acid, Tween 40, and Tween 80. On the other hand, the studied microbiomes differed in the capacity to metabolize glycogen, β-methyl-D-glucoside, α-D-lactose, 2-hydroxybenzoic acid, α-cyclodextrin, and itaconic acid.

### Differences Between Rhizobiomes of *V. virgatum*, *V. corymbosum*, and *V. darrowii*

**FIGURE 4:**
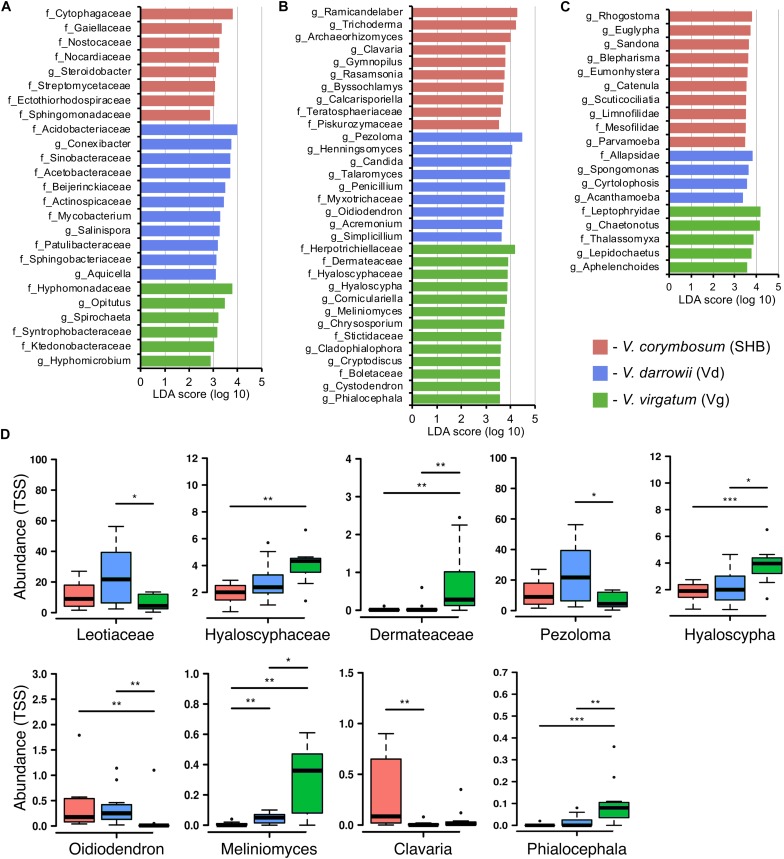
LEfSe analysis of differentially abundant (LDA threshold score ≥ 3.0) families and genera of bacteria **(A)**, fungi **(B)**, and eukaryotes **(C)** between *Vaccinium* species. **(D)** Ericoid mycorrhizal fungi with significant differences (*P ≤ 0.05; **P ≤ 0.01; ***P ≤ 0.001) in the total sum normalized (TSS) transformed abundance.

Differences in the composition of rhizobiomes of different *Vaccinium* species were evaluated at the family and genus level by calculating LEfSe scores that indicate the degree of consistent difference in relative abundance between treatments (Segata et al., 2011). Overall, our results revealed both plant species-level and genotype-level differences in the composition of bacterial, fungal, and eukaryotic communities associated with blueberry roots. A total of 25 distinct bacterial biomarkers were identified using the LDA threshold score of ≥ 3.0, most of which were Proteobacteria (especially *Alphaproteobacteri*a) and *Actinobacteria* (Figure 4A). The SHB-enriched phylotypes belonged to the proteobacterial and actinobacterial families *Ectothiorhodospiraceae*, *Sphingomonadaceae*, *Steroidobacter*, *Gaiellaceae*, *Nocardiaceae*, and *Streptomycetaceae*, as well to the *Cytophagaceae* (*Bacteroidetes*) and *Nostocaceae* (*Cyanobacteria*). The Vd rhizobiome was characterized by the abundance of several *Actinobacteria* (*Conexibacter*, *Actinospicaceae*, *Mycobacterium*, *Salinispora*, and *Patulibacteraceae*) and *Proteobacteria* (*Sinobacteracea*, *Acetobacteraceae*, *Beijerinckiaceae*, and *Aquicella*), and by the presence of *Acidobacteriaceae* (*Acidobacteria*) and *Sphingobacteriaceae* (*Bacteroidetes*). The identified Vg-specific phylotypes were taxonomically diverse and included members of *Proteobacteria* (*Hyphomonadaceae, Syntrophobacteraceae, Hyphomicrobium*), *Verrucomicrobia* (*Opitutus*), *Spirochaetes*, and *Chloroflexi* (*Ktedonobacteraceae*). We also observed genotype-level differences in the distribution of individual families and genera associated with roots of *V. corymbosum* (SHB), *V. darrowii* (Vd), and *V. virgatum* (Vg) (Supplementary Figure S3A).

The analysis of fungal communities revealed 32 distinct biomarkers that were differentially distributed among rhizobiomes of the studied blueberry species (Figure 4B). The rhizobiome of *V. corymbosum* (SHB) was enriched in diverse ascomycetes (*Trichoderma*, *Archaeorhizomyces*, *Rasamsonia*, *Byssochlamys*, and *Teratosphaeriaceae*) and basidiomycetes of the orders *Agaricales* (*Clavaria*, *Gymnopilus*) and *Filobasidiales* (*Piskurozymaceae*). In contrast, the *V. virgatum* (Vg)-specific fungi included an abundance of ascomycetes from the orders *Helotiales*, *Ostropales*, and *Chaetothyriales*, and were particularly enriched in ericoid mycorrhizae (EM) (*Phialocephala*, *Meliniomyces*, *Hyaloscypha*) and endophytic taxa (*Cystodendron*, *Corniculariella*) (Figure 4D). Some closely related EM fungi (i.e., *Pezoloma*, *Oidiodendron*, and *Myxotrichaceae*) were also specifically associated with roots of *V. darrowii* (Vd) along with the members of *Eurotiales* (*Penicillium*, *Talaromyces*), *Hypocreales* (*Acremonium*, *Simplicillium*), and *Candida* yeast (*Saccharomycetales*). We identified multiple fungal taxa that were differentially distributed between genotypes of three *Vaccinium* spp., including several species of EM fungi and plant-beneficial endophytes *Verruconis*, *Gongronella*, *Pestalotiopsis*, and *Cladophialophora* (Supplementary Figure S3B).

**FIGURE 5:**
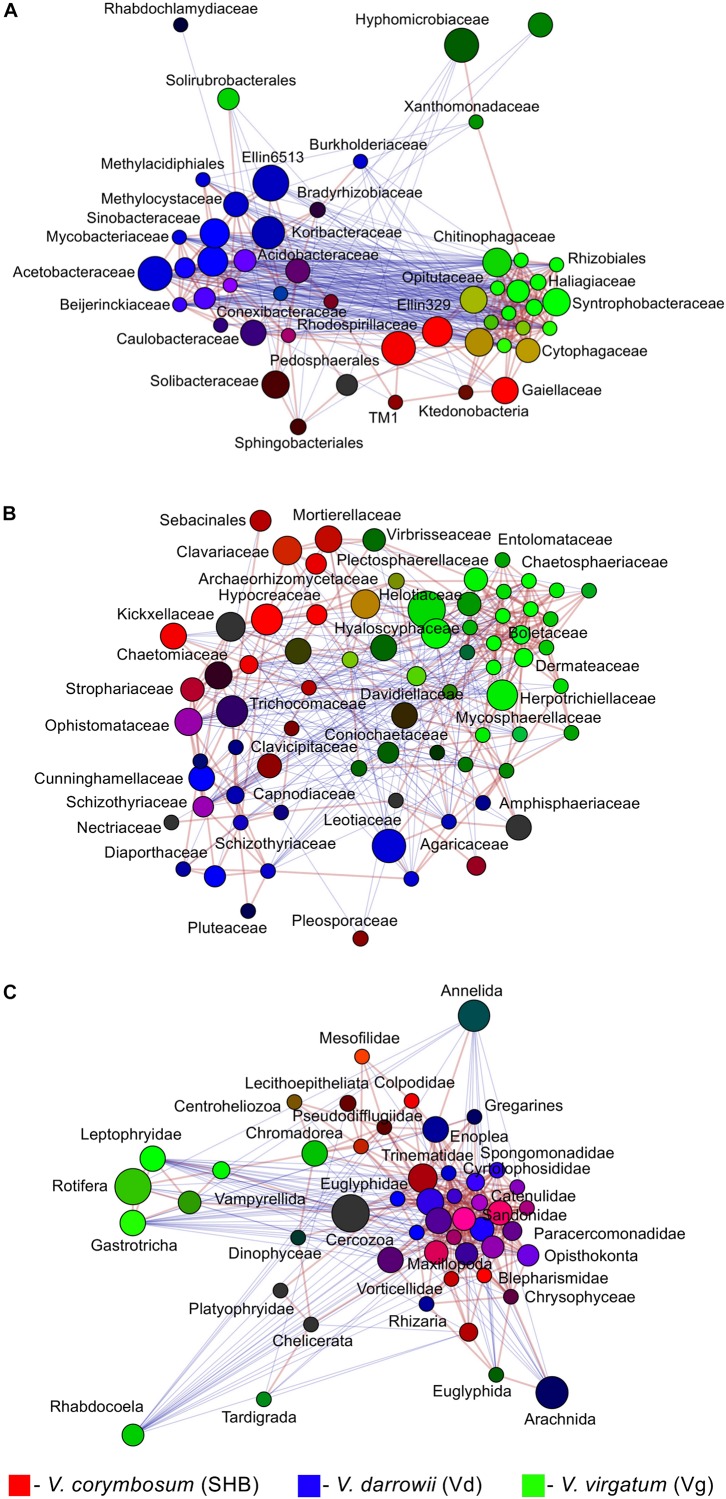
Network analysis illustrating significant co-occurrence patterns among bacterial **(A)**, fungal **(B)**, and eukaryotic **(C)** taxa and *Vaccinium* species. The analysis was performed using Spearman correlations. Network edges represent statistically significant positive (red) and negative (blue) correlations (FDR < 0.05). Taxa are represented by nodes colored based on their association with the studied blueberry species [red, *V. corymbosum* (SHB), blue, *V. darrowii* (Vd), green, *V. virgatum* (Vg)]. The relative brightness (i.e., the color gradient) of each node reflects the significance level of the association.

The LEfSe analysis revealed 19 eukaryotic biomarkers that were differentially enriched in the rhizosphere of *V. corymbosum*, *V. virgatum*, and *V. darrowii* (Figure 4C). Over half of these taxa were represented by amoeboids and flagellates of the phylum *Cercozoa*, which were specifically associated with roots of SHB (*Rhogostoma*, *Euglypha*, *Sandona*, *Limnofilidae*, *Mesofilidae*), Vd (*Allapsidae*, *Spongomonas*), or Vg (*Thalassomyxa*, *Leptophryidae*). Other differentially enriched groups of protists included ciliates (*Blepharisma*, *Scuticociliatia*, *Cyrtolophosis*) and amoebas (*Parvamoeba*, *Acanthamoeba*), which were present in *V. corymbosum* and *V. darrowii*. Finally, 18S-based profiling also identified certain gastrotrichs (*Chaetonotus*, *Lepidochaetus*), microscopic nematodes (*Eumonhystera*, *Aphelenchoides*), and flatworms (*Catenula*) as specific biomarkers associated with SHB and Vg. Although there were fewer genotype-level differences in the distribution of eukaryotic biomarkers compared to bacteria or fungi, we identified several genera of ciliates, filose amoebas, and flagellates that differed in abundance between the two genotypes of *V. corymbosum* (Supplementary Figure S3C). Collectively, the LEfSe results were in agreement with differences in the relative abundance of bacteria, fungi, and eukaryotes estimated by the non-parametric Wilcoxon rank-sum test and visualized in the form of differential heat trees (Supplementary Figure S4). The family-level analysis of rhizosphere microbial communities also revealed the presence of complex co-occurrence networks. The bacterial network contained two distinct clusters of co-occurring taxa (Figure 5A). The first cluster contained some *Actinobacteria* (*Mycobacteriaceae*, *Conexibacteraceae*), *Acidobacteria* (*Acidobacteriaceae*, *Koribacteraceae*), *Alpha-* (*Sinobacteraceae*, *Beijerinckiacea*), and *Gammaproteobacteria* (*Acetobacteraceae*) associated predominantly with *V. darrowii*. The second cluster was associated with *V. virgatum* and was comprised of *Alphaproteobacteria (Rhizobiaceae)*, *Bacteroidetes* (*Chitinophagaceae*, *Cytophagaceae*), *Deltaproteobacteria* (*Syntrophobacteraceae*, *Haliangiaceae*), and *Verrucomicrobia* (*Opitutaceae*). Although the fungal network was overall more diffuse, it still contained a distinct group of co-occurring taxa (*Boletaceae*, *Hyaloscyphaceae*, *Plectosphaerellace*, *Dermateaceae*, *Herpotrichiellacea*, *Chaetosphaeriaceae*, *Helotiaceae*) found in the rhizosphere of *V. virgatum* (Figure 5B). Finally, the eukaryotic network included a large group of positively correlated taxa that were associated with roots of *V. corymbosum* and *V. darrowii*, as well as a much smaller cluster of *Rotifera*, *Gastrotricha*, and *Leptophryidae* that were associated with *V. virgatum* (Figure 5C).

## Discussion

To our knowledge, this is the first study that compares side-by-side rhizosphere microbial communities of SHB (*V. corymbosum* interspecific hybrid), Darrow’s blueberry (*V. darrowii*), and rabbiteye blueberry (*V. virgatum*). Our results revealed that these species share a common core rhizobiome, which is similar in composition to that of the lowbush blueberry *V. angustifolium* (Yurgel et al., 2017). At the same time, the rhizosphere communities of SHB, Vd, and Vg differed significantly in the diversity, relative abundance, richness, and evenness of multiple groups of prokaryotic and eukaryotic microorganisms (Figure 2 and Supplementary Figure S4). Although the host signature effects were especially pronounced at the plant species level, we also observed genotype-level variations in the distribution of specific microbial taxa, which suggests that the assembly of the blueberry microbiome is shaped by the plant genotype and modifications associated with the domestication and breeding of members of the *Vaccinium* genus. Overall, *V. corymbosum* (SHB) and *V. darrowii* (Vd) harbored more similar microbial communities compared to *V. virgatum* (Vg), a finding that aligns well with the fact that southern highbush blueberries were developed by hybridizing the northern highbush blueberry *V. corymbosum* with several other *Vaccinium* species, including *V. darrowii* (Hancock, 2006). Although the impact of plant domestication on the root-associated microorganisms is poorly understood, several recent studies reported that the domestication is associated with compositional changes in the rhizosphere microbiome (Kiers et al., 2007; Zachow et al., 2014; Bulgarelli et al., 2015; Cardinale et al., 2015; Perez-Jaramillo et al., 2016, 2017). Interestingly, a meta-analysis of microbiome studies in barley, lettuce, common bean, and Arabidopsis revealed that members of the *Bacteroidetes* were more abundant in roots of wild plant relatives, while *Proteobacteria* and *Actinobacteria* were consistently associated with the rhizosphere of corresponding domesticated accessions (Perez-Jaramillo et al., 2018). We also identified these phyla among differentially distributed microbial groups and demonstrated that *Bacteroidetes* were associated with *V. virgatum*, whereas *Actinobacteria* were more abundant in *V. darrowii.* However, plants used in this study were grown under controlled conditions. It will be interesting to address the effect of domestication on the blueberry rhizobiome by including in future studies field-grown plants of wild accessions of *V. angustifolium*, *V. darrowii*, *V. arboreum*, *V. myrsinites*, and *V. myrtilloides*.

The analysis of differentially distributed taxa revealed several genera of ericoid mycorrhizal (EM) fungi, which are important mutualistic endosymbionts of blueberries and other plants of the *Ericaceae* family (Grelet et al., 2017). Ericoid mycorrhizae enhance the fitness and productivity of the *Ericaceae* plants and play a vital role in their adaptation to soils with low pH and slow turnover of organic matter. Mycorrhizal fungi supply their host plants with nitrogen, phosphate, and other nutrients, which are mobilized via the breakdown of complex organic compounds by fungal exoenzymes (Kariman et al., 2018). Several studies also reported that EM colonization has positive effects on the tolerance of plants to heavy metals (Daghino et al., 2016). Ericoid mycorrhizae interact with their plant hosts by forming hyphal coils in the epidermal cells of specialized hair roots (Read, 1996). The EM fungi are closely related to Dark Septate Endophytes, a group of fungi with melanized hyphae that form in the root tissue loose loops instead of dense ericoid coils (Vohnik and Albrechtova, 2011). While earlier studies limited the capacity to form ericoid mycorrhizae to a small number of fungi, the advent of molecular methods revealed that hair roots colonized by EM harbor diverse assemblages of ascomycetes of the orders *Helotiales*, *Erysiphales* (*Oidiodendron* spp.), and some basidiomycetes, such as members of the *Sebacinales* and *Clavaria* spp. (Leopold, 2016).

Our results revealed an overall enrichment of the blueberry rhizobiome in various EM fungi, as well as the differential abundance of *Hyaloscyphaceae* (*Hyaloscypha*) and dark septate endophytes (*Meliniomyces*, *Phialocephala*) on roots of *V. virgatum*. We also observed higher levels of *Leotiaceae* (*Pezoloma*) and *Oidiodendron* in *V. darrowii* and an abundance of the EM basidiomycete *Clavaria* in the rhizosphere of *V. corymbosum.* The differential distribution of several EM taxa and plant-beneficial endophytes was also observed at the genotype level. Although the host preference/specificity effects in the interaction of the *Ericaceae* plants with EM fungi are poorly understood, Scagel and Yang (2005) demonstrated that highbush blueberry cultivars that fruit early in the season had higher levels of mycorrhizal infection than cultivars fruiting later in the growing season. Similarly, Sun et al. (2011) demonstrated that communities of EM fungi in *Rhododendron decorum* varied with the host genotype, which was in contrast to non−EM communities that were shaped by geography. That study concluded that genetic make−up of the host plant represents a significant “driving force” shaping communities of ericoid mycorrhizae. Our findings agree with earlier studies that demonstrated the genotype-specific variation in the sensitivity of blueberries to ericoid mycorrhizae and hint at the possibility that the genetics of blueberry plants plays an important role in the recruitment of certain EM fungi from the seed bank of soil microorganisms.

The comparison of rhizosphere communities of SHB, Vd, and Vg revealed several groups of bacteria that may exert beneficial effects on the health and productivity of blueberries. The three compared rhizobiomes had an abundance of free-living diazotrophs from *Bradyrhizobiaceae*, *Methylocystaceae*, *Burkholderiaceae*, and *Frankiaceae*, which may represent another adaptation to poor soils typical of natural habitats of blueberries and other *Ericaceae* plants. The microbiome of *V. darrowii* also had higher levels of nitrogen-fixing *Beijerinckiaceae*, which are often found in the plant rhizosphere where they exchange ammonium for the photosynthetically fixed carbon. We further identified several groups of bacteria that in other crop systems had been implicated in the suppression of soilborne pathogens. For example, the rhizobiome of *V. virgatum* had an increased abundance of *Opitutus*, and the decline of *Opitutaceae* in soil was implicated in the increased susceptibility of cotton to infection with *Fusarium oxysporum* f. sp. *vasinfectum* (Li et al., 2015). The rhizosphere communities of *V. corymbosum* and *V. darrowii* had higher levels of *Sphingobacteriaceae*, *Sphingomonadaceae*, *Cytophagaceae*, *Gaiellaceae*, and several other *Actinobacteria* (Figure 4). *Gaiellaceae* is a recently discovered family that belongs to a deep lineage of the class *Actinobacteria* and consists of a single genus with only one cultured species, *Gaiella occulta* (Albuquerque et al., 2011). These aerobic chemoorganotrophs have been found worldwide in the rhizosphere of corn, rice, soybean, hemp, and sudex (Eo et al., 2015; Sun et al., 2018; Wang et al., 2019). Members of *Gaiellaceae*, together with *Sphingomonadaceae*, and *Streptomycetaceae* were associated with the soil suppressiveness to *F. oxysporum* f. sp. *cubense*, a causative agent of the Panama disease of banana (Xue et al., 2015). The differentially abundant SHB and Vd taxa were also identified among key microbial groups in the Dutch soil suppressive to the plant pathogen *Rhizoctonia solani* AG2-2IIIB (Chapelle et al., 2016). That study employed metagenomic and metatranscriptomic techniques to characterize the transcriptional changes in the rhizobiome of sugar beet plants grown in the presence of the fungal pathogen in a *Rhizoctonia*-suppressive soil. The analysis identified members of the *Sphingobacteriaceae*, *Sphingomonadaceae*, and *Cytophagaceae* among rhizobacteria that responded to the presence of pathogens by upregulation of stress-related genes. A follow-up study by van der Voort et al. (2016) used heat treatment of soil to examine the contribution of different rhizobacterial taxa to the suppression of *R. solani*. The authors suggested that different *Actinobacteria*, including members of the *Streptomycetaceae* and *Mycobacteriaceae*, may contribute to the disease protection since their decrease coincided with the loss of pathogen suppression. Interestingly, *Actinobacteria* were also identified as hub taxa in the rhizosphere microbiome of lowbush blueberry *V. angustifolium*, suggesting their crucial role in the structure of microbial communities of blueberries (Yurgel et al., 2018).

The assembly of rhizosphere microbiome is influenced by the interplay between soil properties (structure, pH, moisture, salinity, organic matter), climate, and human practices (Cordovez et al., 2019). For example, the mycorrhizal colonization in blueberries is modulated by the application of fertilizers, and increased N fertilization can reduce the colonization of roots by ericoid mycorrhizae (Sadowsky et al., 2012). However, given that our study was performed under controlled conditions, it is likely that the observed differences in the structure of rhizosphere microbiomes of the studied *Vaccinium* species are driven by a combination of host effects and microbial interactions. The recruitment of soil microorganisms by plant species and genotypes is mediated by the root morphology and secretion of exudates, which serve as nutrients and signals for the rhizosphere microorganisms (Sasse et al., 2018). A recent elegant study by Zhalnina et al. (2018) demonstrated that the rhizosphere microbiome of wild oat (*Avena barbata*) is recruited via the metabolic synchronization of the root exudation and microbial substrate utilization traits.

Although currently unknown, it is plausible that similar processes govern the assembly of distinct microbial communities in the rhizosphere of different species of blueberry. The observed differences in the composition of microbial communities of *V. corymbosum*, *V. darrowii*, and *V. virgatum* are also likely shaped by mutualistic and competitive interactions between different members of the rhizobiome. The co-occurrence analysis of bacteria, fungi, and protozoa revealed complex networks with numerous positive and negative correlations, similar to those described in the rhizosphere of wild and managed *V. angustifolium* (Yurgel et al., 2018). Interestingly, our analysis also revealed that microbial communities of SHB, Vd, and Vg differ in the abundance of various predatory protists, which feed on bacteria and fungi and have recently emerged as a key factor that shapes the rhizosphere microbiome and selects for plant-beneficial functional traits (Gao et al., 2019).

## Conclusion

Our study is the first to compare the rhizosphere microbial communities of the SHB, Darrow’s blueberry, and rabbiteye blueberry using the modern culture-independent approaches. Our results revealed an extensive diversity of pro- and eukaryotic microorganisms inhabiting the blueberry rhizosphere and demonstrated that the studied species of *Vaccinium* differ in the abundance of beneficial rhizobacteria and EM fungi. Cultivated blueberries were initially selected by crossing several wild relatives to improve the yield and fruit quality. However, the rising popularity of this commodity has expanded the production into areas that present a challenging environment for blueberry plants (Retamales and Hancock, 2018). Blueberries, like other members of the *Ericaceae* family, rely on their microbiomes for the protection against abiotic stresses and survival in soils that are low in nutrients. In particular, mycorrhizal symbiotic partners improve the uptake of soil nutrients, efficacy of fertilizers, and protect plants from the metal toxicity in acidic soils (Scagel, 2005; Scagel and Yang, 2005; Vega et al., 2009; Caspersen et al., 2016). Therefore, we suggest that species- and genotype-specific differences in the structure and function of rhizobiome represent an important facet in the adaptation of blueberry cultivars to soil and climate conditions.

The rhizosphere microbiome is shaped by exudates and root morphology, both of which are influenced by plant genotype (Sasse et al., 2018). There is a growing consensus that the traditional plant breeding should be expanded to include plant genotype × environment × microbiome interactions (Wei and Jousset, 2017). Such microbiome-supported breeding should be performed in the absence of excessive fertilizers and pesticides and complemented by the microbial community profiling and screening for metabolites that mediate interactions with key pathogenic or beneficial species (Wille et al., 2018). We reason that similar experimental approaches can be employed in blueberries to harness microbial communities that improve the disease resistance, tolerance to heat and drought, and vigor to thrive in soils with low organic matter content.

## Data Availability Statement

Sequences generated in this project were deposited in the NCBI sequence read archive under accession numbers PRJNA577971 and PRJNA578171.

## Author Contributions

DM, OM, and EB conceived the research project. EB provided blueberry plants. OM and JL extracted soil DNA. OM and CB conducted the Biolog EcoPlate profiling. DM, JL, OM, and JH conducted the microbiome analysis. DM, JL, OM, and EB wrote the manuscript. All authors contributed to the manuscript revision.

## Conflict of Interest

The authors declare that the research was conducted in the absence of any commercial or financial relationships that could be construed as a potential conflict of interest.

## Footnotes

1 http://cgeb-imr.ca/

2 http://www.bioinformatics.babraham.ac.uk/projects/fastqc

3 http://hannonlab.cshl.edu/fastx_toolkit/download.html
