# Supplementary material for: Comparative Analysis of Rhizosphere Microbiomes of Southern Highbush Blueberry (Vaccinium corymbosum L.), Darrow’s Blueberry (V. darrowii Camp), and Rabbiteye Blueberry (V. virgatum Aiton)
Source: Front Microbiol. 2020 Mar 12;11:370. doi: 10.3389/fmicb.2020.00370 (PMC7081068; doi:10.3389/fmicb.2020.00370)
Supplement: Supplementary file 1 [file Data_Sheet_1.PDF]

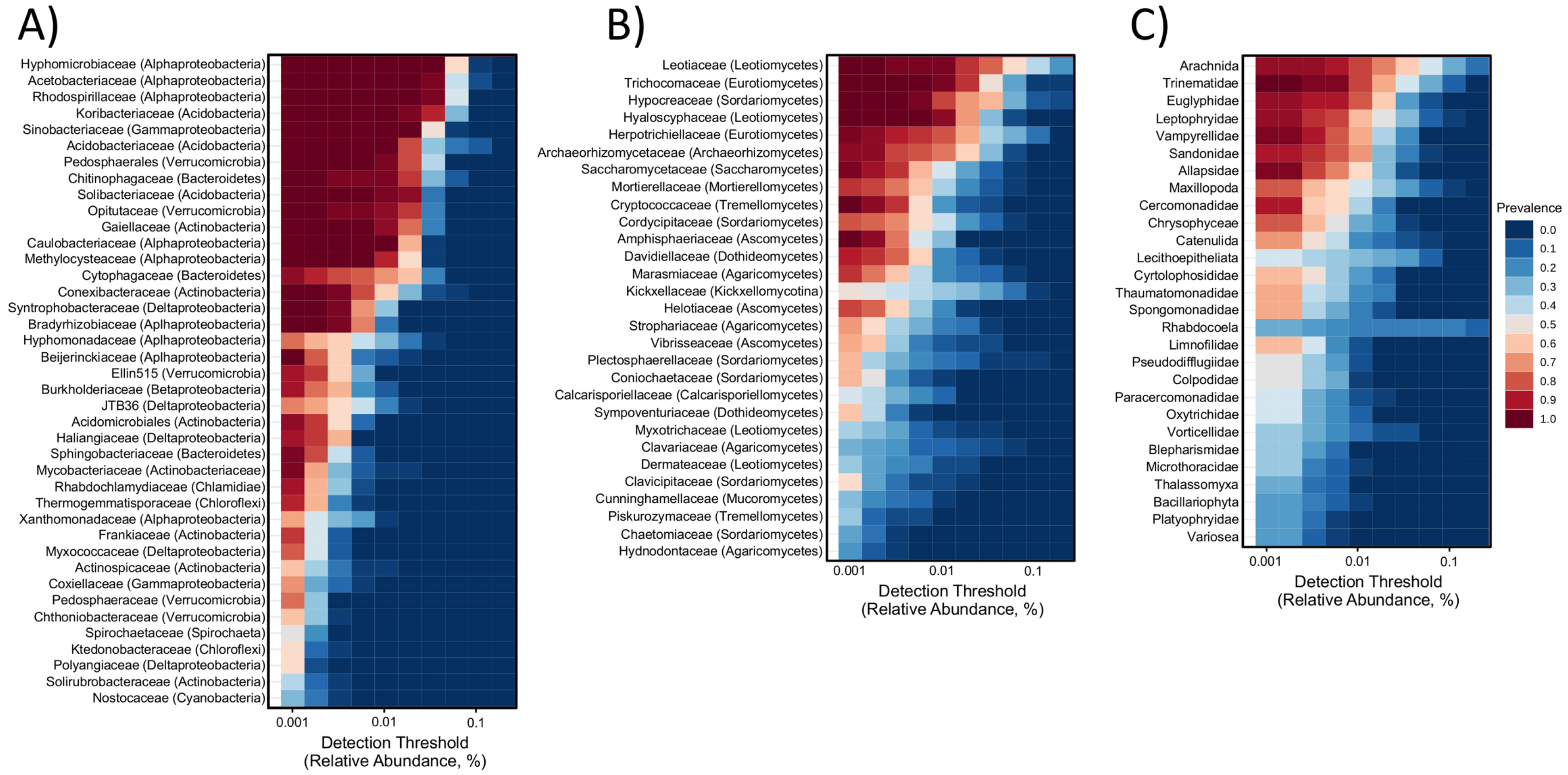

Figure S1. Core microbiome heatmaps illustrating the abundance and prevalence of bacterial (A), fungal (B), and eukaryotic taxa (C) across the studied *Vaccinium* species.

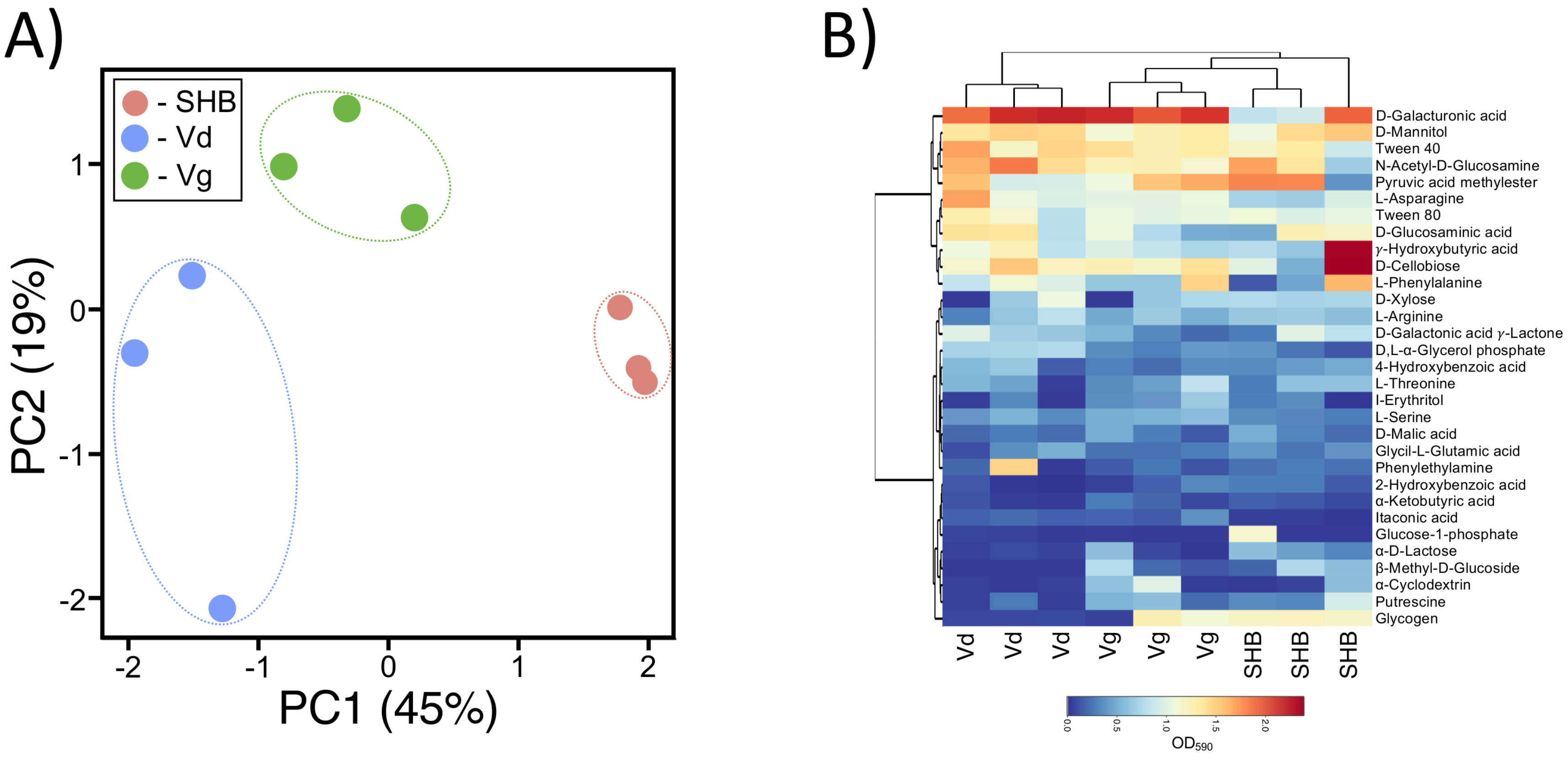

Figure S2. Principal component analysis (A) and hierarchical clustering (B) of metabolic profiles of *Vaccinium* rhizobiomes. The utilization of 31 carbon substrates present in the Biolog EcoPlate is shown by the heatmap.

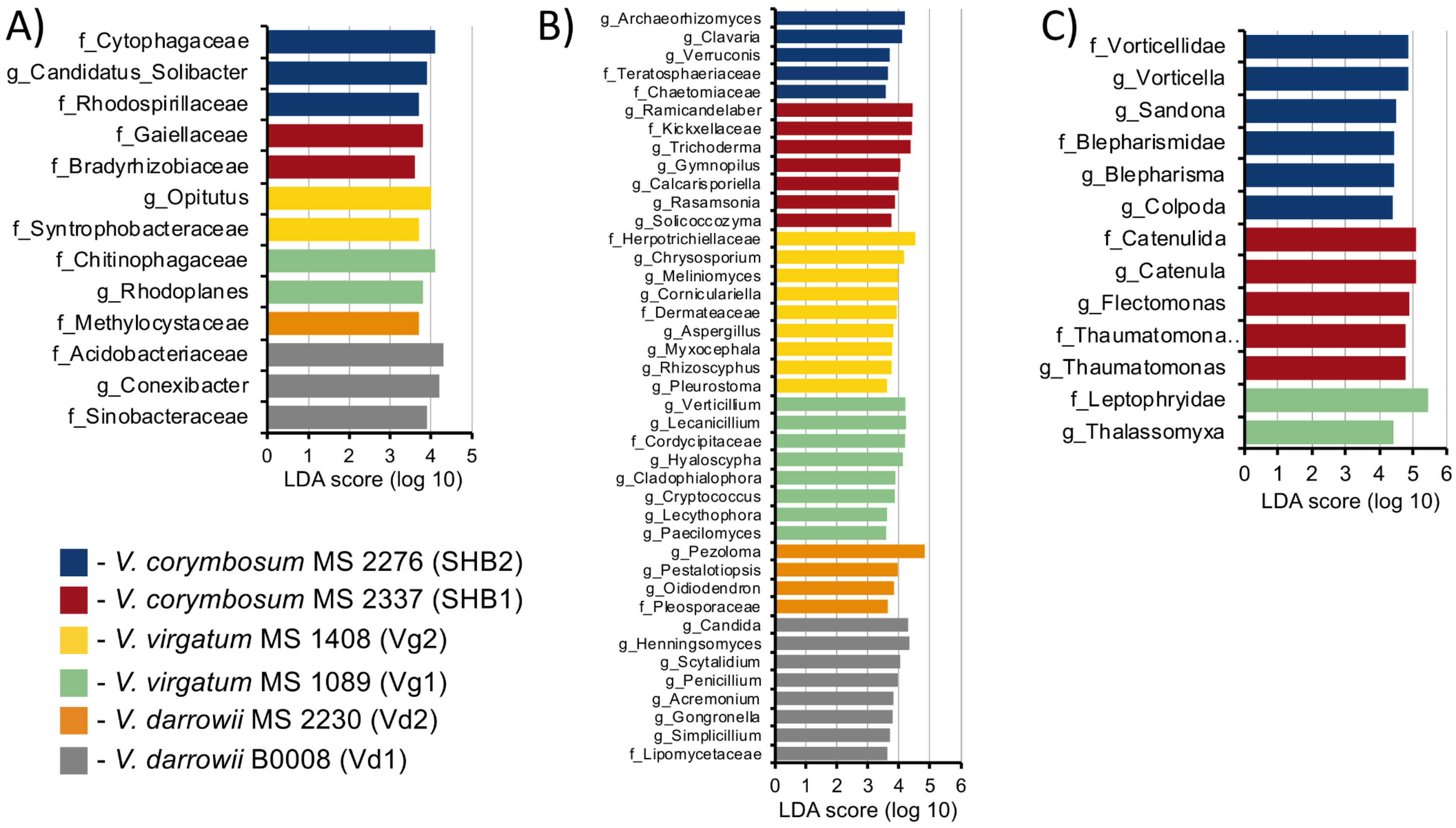

Figure S3. LEfSe analysis of differentially abundant (LDA threshold score ≥3.0) families and genera of bacteria (A), fungi (B), and eukaryotes (C) between different cultivars (genotypes) of *V. corymbosum*, *V. darrowii*, and *V. virgatum*.

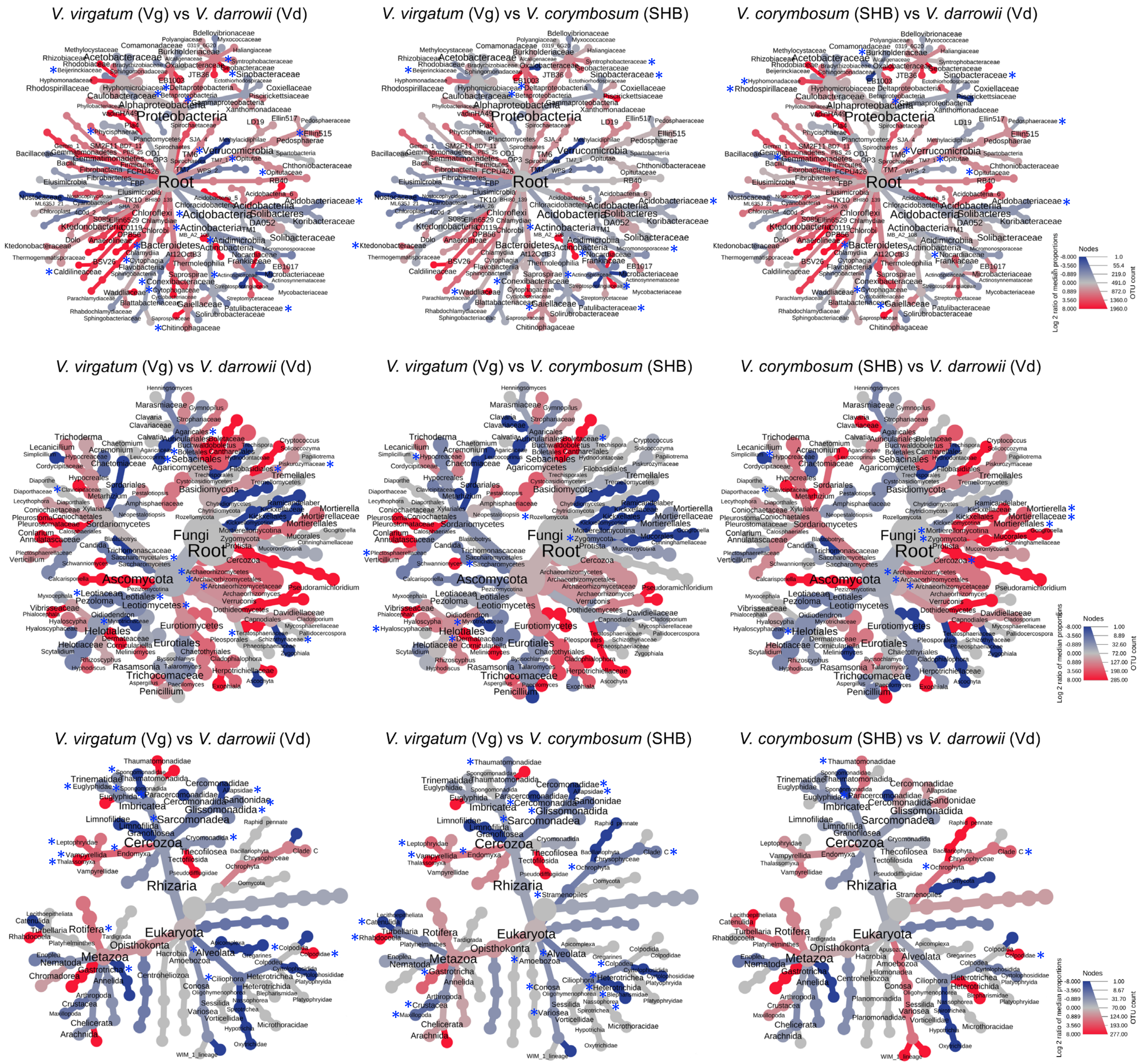

Figure S4. Differential heat trees of pairwise comparisons between bacterial (top row), fungal (medium row), and eukaryotic (bottom) communities of the studied *Vaccinium* species. The node size and color reflect the relative proportion for each taxon based on the log-2 ratio of median proportions of reads observed in the compared pair of rhizobiomes. Taxa highlighted with asterisks are differentially abundant according to Wilcoxon rank-sum test ($P < 0.05$).

Supplementary Table 1. ANOSIM analysis of bacterial, fungal, and eukaryotic communities in the rhizosphere of *V. corymbosum* (SHB), *V. darrowii* (Vd), and *V. virgatum* (Vg).

Species or cultivars of *Vaccinium*	Community	ANOSIM [a]	
		R value	*P* value
Vg, SHB, Vd species	Bacterial	0.605	0.001
SHB1, SHB2 genotypes	Bacterial	0.487	0.006
Vd1, Vd2 genotypes	Bacterial	0.698	0.008
Vg1, Vg2 genotypes	Bacterial	0.432	0.010
Vg, SHB, Vd species	Fungal	0.613	0.001
SHB1, SHB2 genotypes	Fungal	0.544	0.005
Vd1, Vd2 genotypes	Fungal	0.963	0.004
Vg1, Vg2 genotypes	Fungal	0.474	0.011
Vg, SHB, Vd species	Eukaryotic	0.548	0.001
SHB1, SHB2 genotypes	Eukaryotic	1.000	0.200
Vd1, Vd2 genotypes	Eukaryotic	0.694	0.014
Vg1, Vg2 genotypes	Eukaryotic	0.302	0.061

[a]Permutational multivariate analysis of variance (PERMANOVA) test using a Bray-Curtis dissimilarity matrix with 999 permutations.
